# Heparin-like effect of a dual antiplatelet and anticoagulant (APAC) agent on red blood cell deformability and aggregation in an experimental model

**DOI:** 10.1007/s11239-024-03040-8

**Published:** 2024-09-04

**Authors:** Adam Attila Matrai, Adam Varga, Barbara Bedocs-Barath, Erzsebet Vanyolos, Rita Orban-Kalmandi, Linda Loczi, Zsuzsa Bagoly, Annukka Jouppila, Riitta Lassila, Norbert Nemeth, Adam Deak

**Affiliations:** 1 Department of Operative Techniques and Surgical Research, Faculty of Medicine, University of Debrecen, Moricz Zsigmond u. 22, Debrecen, H-4032 Hungary; 2 Division of Clinical Laboratory Science, Department of Laboratory Medicine, Faculty of Medicine, University of Debrecen, and Hungarian Research Network (HUN-REN-DE) Cerebrovascular Research Group, Nagyerdei krt. 98, Debrecen, H-4032 Hungary; 3 Helsinki University Hospital Clinical Research Institute, Tukholmankatu 8, Helsinki, FI-00290 Finland; 4 Coagulation Disorders Unit, Departments of Hematology and Comprehensive Cancer Center and Research Program Unit in Systems Oncology, Helsinki University Hospital, University of Helsinki, Haartmaninkatu 4, Helsinki, FI- 00290 Finland

**Keywords:** Antiplatelet, Anticoagulant, Hemorheology, Red blood cell deformability, Red blood cell aggregation

## Abstract

Treatments with different antithrombotic agents can affect micro-rheological variables, such as red blood cell (RBC) deformability and aggregation. Since the effect of dual antiplatelet and anticoagulant (APAC) treatment on micro-rheology is unknown, we aimed to investigate the effect of different intravenous doses of APAC on hematological and micro-rheological variables in a porcine model. Two groups were formed (APAC group, Control group), and blood was collected from the animals at preset intervals. Hematological variables, RBC deformability, and aggregation were measured. We observed an improvement in the RBC deformability measured at a low shear stress range (< 3 Pa). However, after both doses, a decrease in the maximal elongation index of RBC values occurred in the APAC group. RBC aggregation increased after APAC bolus dose, while it gradually and dose-dependently decreased. Supposedly, the improvement in RBC deformability that was observed at a lower shear rate could facilitate aggregation. Administration of APAC and unfractionated heparin (UFH) caused comparable changes in hematological and hemorheological variables. Signs of thrombosis or bleeding did not occur. APAC and UFH had comparable micro-rheological effects.

## Introduction

Hemorheological properties, including red blood cell (RBC) deformability and aggregation, may be altered in numerous pathophysiological cases influenced by several preanalytical factors, including temperature variation, sampling technique, sample storage and the anticoagulant of the blood collection tubes [1–6]. Anticoagulants may induce distinct changes in hemorheological variables.

Unfractionated heparin (UFH), a common clinical antithrombotic agent, has been reported to increase red blood cell sedimentation rate and whole blood viscosity at low shear rates, suggesting an increase in red blood cell aggregation [7]. Despite his property, heparin is used in cannulas during many intravascular procedures, but unfractionated heparin (UFH) is also the current international standard anticoagulant during extracorporeal membrane oxygenation and cardiovascular surgery [8].

The excess of RBC aggregation may obstruction small blood vessels, particularly those in the microcirculation, which lowers the amount of oxygen and nutrients that reach tissues [9–11]. Blood’s ability to thinning under shear is mostly determined by RBC aggregation, which raises blood viscosity as shear rates decrease. Reduced nitric oxide generation and altered vascular tone may result from this [12].

Heparin is derived from mast cells in form of heparin proteoglycans, in the same location on the blood vessel walls as tissue factor. During tissue damage, mast cells are activated and release heparin proteoglycans (HEP-PG) with a higher molecular weight than UFH [13–15] with antiplatelet (towards collagen and thrombin) and anticoagulant properties [13, 15]. We have developed a dual AntiPlatelet and AntiCoagulant, APAC, which mimics the naturally occurring HEP-PGs to reach antithrombotic and anti-inflammatory activities, upon bioconjugation of UFH to albumin carrier [16, 17]. In citrated human plasma or whole blood in vitro, APAC, in a concentration-dependent manner, inhibits collagen-induced platelet aggregation [14, 18], attenuates coagulation initiated by intrinsic pathways, prolongs the activated partial thromboplastin time (APTT), and in whole blood reduces platelet and fibrin deposition on thrombogenic surfaces in a von Willebrand factor (VWF)-dependent mechanism [19].

In porcine, baboon, and mouse thrombosis models, APAC acts as an antithrombotic agent by colocalizing with VWF and laminin at the fresh injury site [14, 20]. In a rat model of ischemic acute kidney injury, APAC shows vasculoprotective effects [21]. The complexity of cardiovascular disease has necessitated the combined use of antithrombotic agents with different mechanisms - antiplatelet, anticoagulant, and fibrinolytic agents [19, 22, 23]. The drugs may enhance efficacy, but the risk of hemorrhage increases with the number of drugs used [19, 24, 25]. Therefore, the use of a single antithrombotic formulation, having both selected antiplatelet and anticoagulant functions may have a major impact in this area [19, 25].

The aim of this study was to investigate the effect of APAC on hemorheological variables. We investigated whether APAC would also affect RBC micro-rheology and microcirculation. It was hypothesized that the dual anti-platelet and anticoagulant formulation would have an effect similar to UFH in the micro-rheological variables of RBC and compared APAC with UFH on their micro-rheological properties.

## Materials and methods

### Experimental animals and sampling protocol

#### Hemorheological measurements

**Fig. 1:**
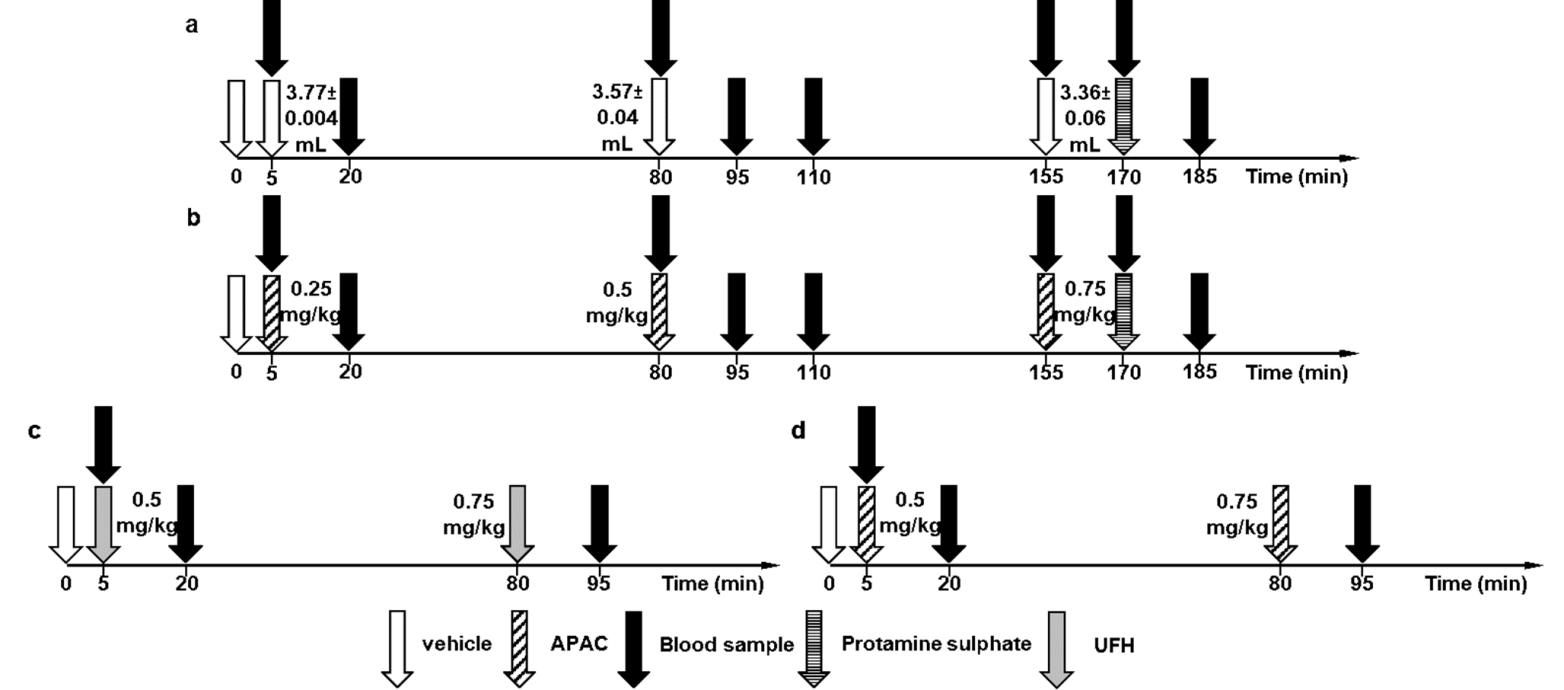
Sampling times and intravenous bolus administration times in the Control group (*n* = 5) (**a**) and APAC group (*n* = 5) (**b**). Times of administration of the different bolus doses of UFH and APAC and blood sampling in the UFH group (*n* = 5) (**c**) and APAC group (*n* = 5) (**d**). Open arrow refers to vehicle (1 mL of 137 mM NaCl and 10 mM Na_2_HPO_4_, pH: 7.5), hatched arrow to APAC, black arrow refers to blood sampling, lined arrow to protamine sulphate and grey arrow to UFH

Venesection (external jugular veins) was performed under general anesthesia (pre-medication: 2 mg/kg azaperone (Stresnil, i.m.; induction of anesthesia: 2 mg/kg xylazine (CP-Xylazine-hydrochloride, 2%; Produlab Pharma BV, The Netherlands), i.m., 20 mg/kg ketamine (CP-Ketamine hydrochloride 10%, Produlab Pharma BV, The Netherlands), maintenance of anesthesia: 0.9 mg/kg xylazine and 8 mg/kg ketamine. Blood was drawn (ethical approval registered by University of Debrecen Committee of Animal Welfare, Nr.: 3/2021/UDCAW) from the right external jugular vein of female Hypor pigs (*n* = 5/group; bodyweight: 23.6 ± 1.58 kg) to the vacutainer tubes (BD Vacutainer^®^ tubes, 1.8 mg/ml K_3_-EDTA; Becton, Dickinson and Company, USA), and the vehicle (1 mL of 137 mM NaCl and 10 mM Na_2_HPO_4_, pH: 7.5) and test substance (APAC; 7.84 mg/ml; Aplagon Ltd/Cadila Pharmaceuticals Ltd.) was group-specifically injected intravenously (i.v.) via the left external jugular vein. After cannulation, a continuous fluid replacement was provided on both sides (right and left external jugular vein) during the study with physiological saline (1080.4 ± 45.9 ml, “Baxter” Sodium Chloride 0.9%, pH = 4.5-7, osmolarity: 308 mOsm/l, Baxter Hungary Kft.). For urine drainage, a suprapubic cystostomy catheter was inserted. A tube was placed in the trachea to assist ventilation (WATO EX-20Vet, Shenzhen Mindray Animal Medical Technology Co., LTD., China). PaCO_2_ was set to 35–45 mmHg, and PaO_2_ was set at 100–130 mmHg. The study protocol is illustrated in parts *a* and *b* of Fig. 1.

At the beginning of the study, the vehicle (1 mL of 137 mM NaCl and 10 mM Na_2_HPO_4_, pH: 7.5) was introduced for both groups, followed by blood sampling 5 min later. Subsequently, APAC was administered at escalating doses of 0.25 mg/kg, 0.5 mg/kg, and 0.75 mg/kg, i.v. The current estimated clinical IV bolus dosing is compatible with the doses of 0.25–0.5 mg/kg (approx. 45–90 IU/kg of heparin), whereas 0.75 mg/kg is over the maximal allowance based on the toxicology program [26]. The Control group was administered only the vehicle only. The volume of the formulations containing the active substance was 4 mL, of which 3.77 ± 0.004 mL was the volume of vehicle administered to the control animals for the 0.25 mg/kg dose 3.57 ± 0.04 mL, for the 0.5 mg/kg and 3.36 ± 0.06 mL for the 0.75 mg/kg dose. For both groups, blood was collected 15 min after the first administration. At the end of the study, 140 IU/kg protamine sulphate (1400 anti-heparin IU/ml, lot F2084FI1, Leo Pharma, Denmark) was administered, and blood was taken again 15 min later. The blood samples were collected also for a cooperative coagulation biomarker study.

#### Comparison of APAC with UFH

The anesthesia of the animals was the same as previously described. Blood was drawn from the right external jugular vein of female Hypor pigs (*n* = 5/group; bodyweight: 23.3 ± 1.06 kg) to the vacutainer tubes (BD Vacutainer^®^ tubes, 1.8 mg/ml K_3_-EDTA; Becton, Dickinson and Company, USA), and the vehicle (1 mL of 137 mM NaCl and 10 mM Na_2_HPO_4_, pH: 7.5) and APAC or UFH (Heparibene Na 25000 IU, Ratiopharm Arzneimittel Vertriebs GmbH.) were injected i.v. through the left vein. The effects of preparations at 0.5 mg/kg (the maximal clinical APAC dose, approx. 90 IU/kg of heparin i.v.) and 0.75 mg/kg were compared. The study protocol is illustrated in parts *c* and *d* of Fig. 1.

The vehicle was administered first, followed by the low dose, and then escalated with the higher bolus dose of APAC or UFH. The first blood sample was obtained 5 min after the vehicle was administered and was taken as a baseline; the blood was collected again 15 min after the test substances were administered.

### Hematological variables

The qualitative and quantitative hematological variables were measured by ADVIA 120 hematology automate (Siemens Healthcare Gmbh, Germany) and Sysmex K-4500 microcell counter (TOA Medical Electronics Co., Ltd., Kobe, Japan). In this study, red blood cell count (RBC [T/L]), white blood cell count (WBC [G/L]), hemoglobin concentration (Hgb [g/dl]), hematocrit (Hct [%]), mean corpuscular volume (MCV [fl]), mean corpuscular hemoglobin (MCH [pg]), mean corpuscular hemoglobin concentration (MCHC [g/dl]), and platelet count (Plt [G/L]) were assessed.

### Red blood cell deformability measurements

RBC deformability was tested using a LoRRca MaxSis Osmoscan ektacytometer (Mechatronics BV, The Netherlands) [27]. For the deformability test 10 µl whole blood were gently dispersed in 2 ml of polyvinylpyrrolidone (PVP)–PBS solution (PVP: 360 kDa, Sigma-Aldrich Co. USA; PVP-PBS solution viscosity = 30.4 mPas, osmolality = 302 mOsmol/kg, pH = 7.2). In the device elongation index (EI) was determined in the function of shear stress (SS [Pa], range: 0.3–30 Pa) [28]. For the comparison of the EI-SS curves EI values at 3 Pa, maximal elongation index (EImax), and the shear stress belonging to the half EImax (SS1/2, [Pa]) were used, based on Lineweaver-Burk equation [29].

### Determination of red blood cell aggregation

Using a LoRRca MaxSis Osmoscan ektacytometer (Mechatronics BV, The Netherlands), the RBC aggregation was examined. The laser backscattering method was used to run the device. The Couette-system rotates the blood sample to be disaggregated; the rotor then quickly stops, allowing the variations in the light’s intensity reflection from the blood sample to be detected [27, 28]. Aggregation index (AI [%]) was the parameter that was analyzed. The test needs one milliliter of whole blood.

### Statistical analysis

SigmaStat Software 3.1.1.0 (Systat Software Inc., San Jose, CA, USA) was used to carry out the statistical analyses. Data are expressed as mean ± S.D. (standard deviation). The number of cases was estimated using the statistical program G*power. Differences between groups were analyzed by t-test or the Mann–Whitney rank-sum test, differences between each blood sampling by paired t-test or Wilcoxon rank sum test, and the repeated measures ANOVA or Friedman’s test was used based on the results of the Kolmogorov–Smirnov normality test. A p-value of < 0.05 was considered statistically significant.

## Results

### Hemorheological effects of APAC

#### Hematological variables

Overall, the complete blood count (CBC) did not change significantly despite some minor variation. All values remained in the biologically normal range throughout the experiment. Slight changes in hematological variables were observed after each dose, but in some cases these changes were statistically significant (Hct: *p* = 0.008, Plt: *p* = 0.031), but in all cases the values were biologically within the normal range. Top of Table 1 shows the changes of selected hematological variables of whole blood samples collected according to the experimental protocol.

**Table 1:** Hematological variables in control group (*n* = 5) and APAC group (*n* = 5) along the escalation dose scheme (measured by ADVIA 120 hematology automate) (above)

Sampling time	Group	RBC (T/L)	Hgb (g/L)	Hct (%)	MCV (fL)	Plt (G/L)
5 min after vehicle	Control	6.16 ± 0.28	95.4 ± 5.0	32.66 ± 1.29	53.04 ± 0.91	478.80 ± 49.06
	APAC	5.70 ± 0.37	89.4 ± 5.6	29.22 ± 1.75#	52.82 ± 2.08	458.20 ± 57.68
75 min after 0.25 mg/kg	Control	6.19 ± 0.35	96.2 ± 6.8	32.28 ± 2.10	52.22 ± 0.99	481.80 ± 56.15
	APAC	5.69 ± 0.57	92.4 ± 6.3	29.94 ± 2.32	52.80 ± 1.90	462.80 ± 66.09
75 min after 0.5 mg/kg	Control	6.42 ± 0.29	100.2 ± 4.1	33.54 ± 1.30	52.24 ± 0.95	485.30 ± 35.00
	APAC	6.06 ± 0.51	97.6 ± 7.0	31.92 ± 2.23	52.82 ± 2.00	443.00 ± 54.08
15 min after protamine sulphate	Control	6.57 ± 0.28	102.0 ± 4.0	34.30 ± 1.17	52.20 ± 1.01	490.80 ± 37.24
	APAC	6.15 ± 0.55	98.0 ± 7.8	32.30 ± 2.65	52.60 ± 2.10	432.00 ± 18.92#
Sampling time	Group	EI at 3 Pa	EI_max_	SS_1/2_ (Pa)	EI_max_/SS_1/2_	
5 min after	Control	0.33 ± 0.01	0.53 ± 0.04	1.95 ± 0.41	0.28 ± 0.04	
vehicle	APAC	0.34 ± 0.02	0.55 ± 0.02	1.98 ± 0.40	0.28 ± 0.05	
75 min after	Control	0.34 ± 0.02	0.53 ± 0.02	1.75 ± 0.29	0.31 ± 0.05	
0.25 mg/kg	APAC	0.35 ± 0.01	0.53 ± 0.02	1.75 ± 0.27	0.31 ± 0.04	
75 min after 0.5 mg/kg	Control	0.35 ± 0.01	0.55 ± 0.01	1.67 ± 0.16	0.33 ± 0.03	
	APAC	0.36 ± 0.02	0.53 ± 0.02#	1.58 ± 0.39	0.35 ± 0.06	
15 min after protamine sulphate	Control	0.35 ± 0.01	0.54 ± 0.01	1.69 ± 0.24	0.33 ± 0.05	
	APAC	0.35 ± 0.02	0.52 ± 0.03*#	1.55 ± 0.28*	0.34 ± 0.05*	

RBC: red blood cell count; Hgb: hemoglobin; Hct: hematocrit; MCV: mean corpuscular volume; Plt: platelet count; EI at 3 Pa: elongation index at 3 Pa; EI_max_: calculated maximal elongation index; SS_1/2_: shear stress at half EI_max_ and EI_max_/SS_1/2_ ratio. Data are presented as mean ± S.D.; *p* < 0.05; * vs. 5 min after vehicle; # vs. Control

Red blood cell deformability results in Control group (*n* = 5) and APAC group (*n* = 5) blood samples (below)

#### Red blood cell deformability

**Fig. 2:**
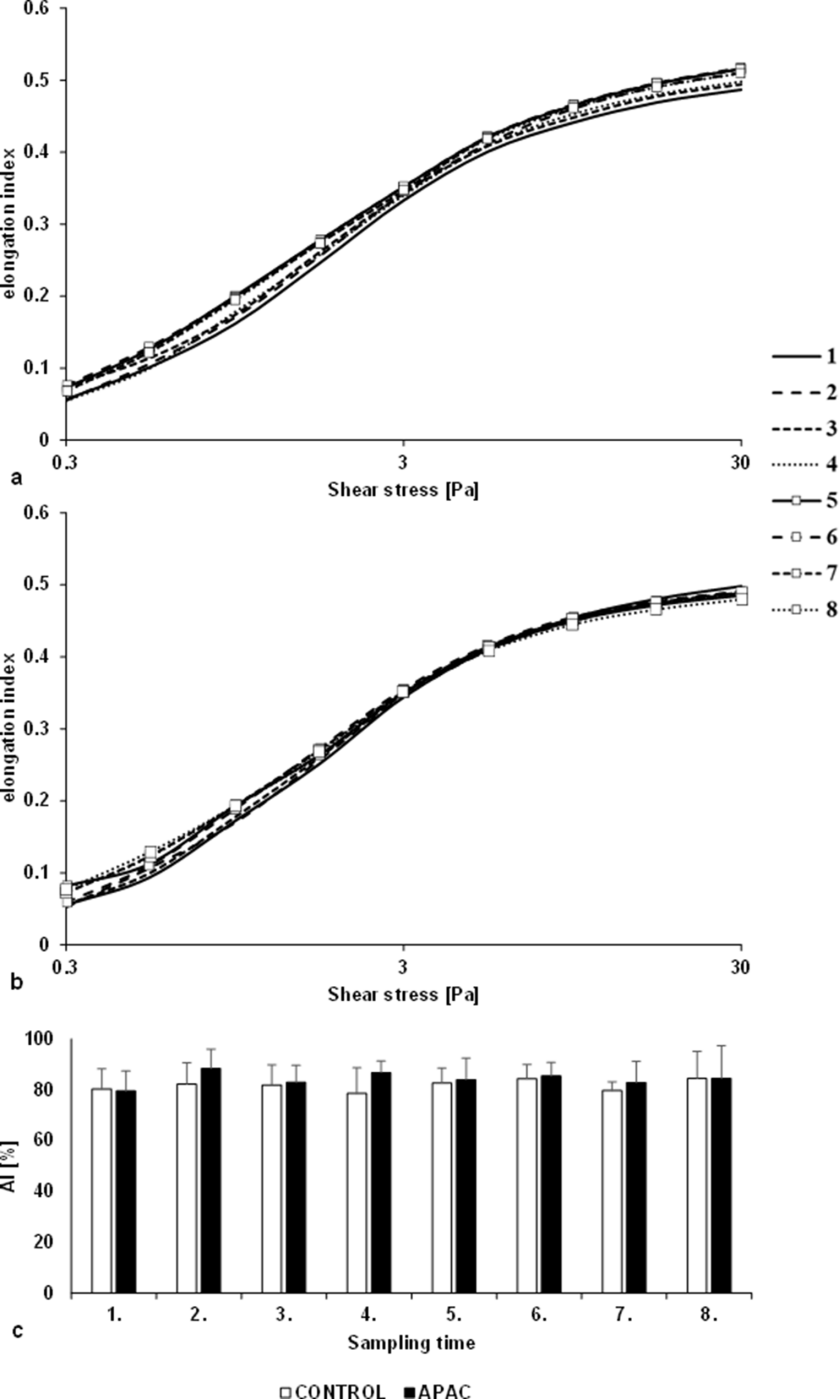
Elongation index in the function of shear stress in Control group (**a**) (*n* = 5) and APAC group (**b**) (*n* = 5) blood samples. Aggregation index (AI [%]) values measured after disaggregation (**c**). Blood sampling times (1) 5 min after administration of the vehicle; (2) 15 min after the first dose; (3) 75 min after the first dose; (4) 15 min after the second dose; (5) 30 min after the second dose; (6) 75 min after the second dose; (7) 15 min after the third dose; (8) 15 min after protamine sulphate

For the EI-SS curves, the elongation index values at 3 Pa, maximal EI and the SS belonging to the half maximal EI were compared, based on Lineweaver-Burk equation: 1/EI = SS_1/2_/EI_max_ x 1/SS + 1/EI_max_. The EI values at the SS of 3 Pa did not change significantly in any of the groups in parts *a* and *b* of Fig. 2.

Comparing the data obtained by parameterizing the curves, it was observed that at a higher shear stress (30 Pa), the maximal EI values impaired in the treated group. 75 min after the 0.5 mg/kg dose, these decreases reached significance compared to the Control group (*p* = 0.024). In bottom of Table 1. some parameters were significantly reduced in the APAC group 15 min after administration of protamine sulphate (EI_max_: *p* = 0.032 vs. Control group; EI_max_: *p* = 0.047, SS_1/2_: *p* = 0.026, EI_max_/SS_1/2_: *p* = 0.034 vs. 5 min after vehicle).

#### Red blood cell aggregation

In the RBC aggregation based on light reflection, differences in the aggregation index values were not observed between the groups when measured under static conditions after disaggregation. A slight increase in the aggregation index was observed in the APAC group after administration of each dose compared to the Control group, but this slight increase was not significant, as observed in part *c* of Fig. 2.

### Comparison of APAC with UFH

#### Hematological parameters

The slight hemoconcentration seen in the APAC group was also observed in the UFH group after both doses. A relative increase in platelet count was detected in the UFH group (0.5 mg/kg *p* = 0.01 vs. baseline). In contrast, a relative decrease of platelet count was found in the APAC group after both doses (*p* < 0.001 vs. baseline). Although some hematological values were significantly altered, they were all biologically within the normal laboratory range for the species [30].

The selected quantitative and qualitative hematological variables are summarized in top of Table 2.

**Table 2:** Hematological parameters in UFH group (*n* = 5) and APAC group (*n* = 5) blood samples (measured by Sysmex K-4500 microcell counter) (above)

Group		RBC (T/L)	Hgb (g/L)	Hct (%)	MCV (fL)	Plt (G/L)
baseline	UFH	5.62 ± 0.18	91.1 ± 6.8	31.37 ± 1.70	55.61 ± 2.77	359.00 ± 22.92
	APAC	5.64 ± 0.46	90.8 ± 2.0	31.53 ± 0.33	55.01 ± 0.65	457.50 ± 24.30
0.5 mg/kg	UFH	5.88 ± 0.19*	94.1 ± 7.6*	32.20 ± 1.71*	55.95 ± 2.54	382.33 ± 31.72*
	APAC	6.02 ± 0.42*	97.4 ± 2.9*	34.38 ± 0.69*	55.81 ± 0.74*	361.13 ± 13.17*
0.75 mg/kg	UFH	5.80 ± 0.32	96.0 ± 9.7*	33.27 ± 2.07*#	56.19 ± 2.84*	381.67 ± 40.50
	APAC	6.23 ± 0.48*#	101.5 ± 5.3*#	35.58 ± 0.96*#	56.14 ± 0.72*#	364.00 ± 19.99*
Group		EI at 3 Pa	EI_max_	SS_1/2_ (Pa)	EI_max_/SS_1/2_	
baseline	UFH	0.34 ± 0.02	0.54 ± 0.01	1.75 ± 0.28	0.31 ± 0.05	
	APAC	0.34 ± 0.02	0.55 ± 0.02	1.98 ± 0.40	0.28 ± 0.05	
0.5 mg/kg	UFH	0.35 ± 0.02	0.54 ± 0.01	1.65 ± 0.32	0.33 ± 0.06	
	APAC	0.35 ± 0.02	0.53 ± 0.01*	1.72 ± 0.24*	0.31 ± 0.05	
0.75 mg/kg	UFH	0.36 ± 0.01*	0.54 ± 0.01	1.64 ± 0.31	0.34 ± 0.05*	
	APAC	0.35 ± 0.02	0.53 ± 0.02*+	1.60 ± 0.29*	0.34 ± 0.06	

RBC: red blood cell count; Hgb: hemoglobin; Hct: hematocrit; MCV: mean corpuscular volume; Plt: platelet count. EI at 3 Pa: elongation index at 3 Pa; EI_max_: calculated maximal elongation index; SS_1/2_: shear stress at half EI_max_ and EI_max_/SS_1/2_ ratio. Data are presented as mean ± S.D.; *p* < 0.05; * vs. baseline; # vs. 0.5 mg/kg; + vs. UFH

Red blood cell deformability results in UFH group (*n* = 5) and APAC group (*n* = 5) blood samples (below)

#### Red blood cell deformability

**Fig. 3:**
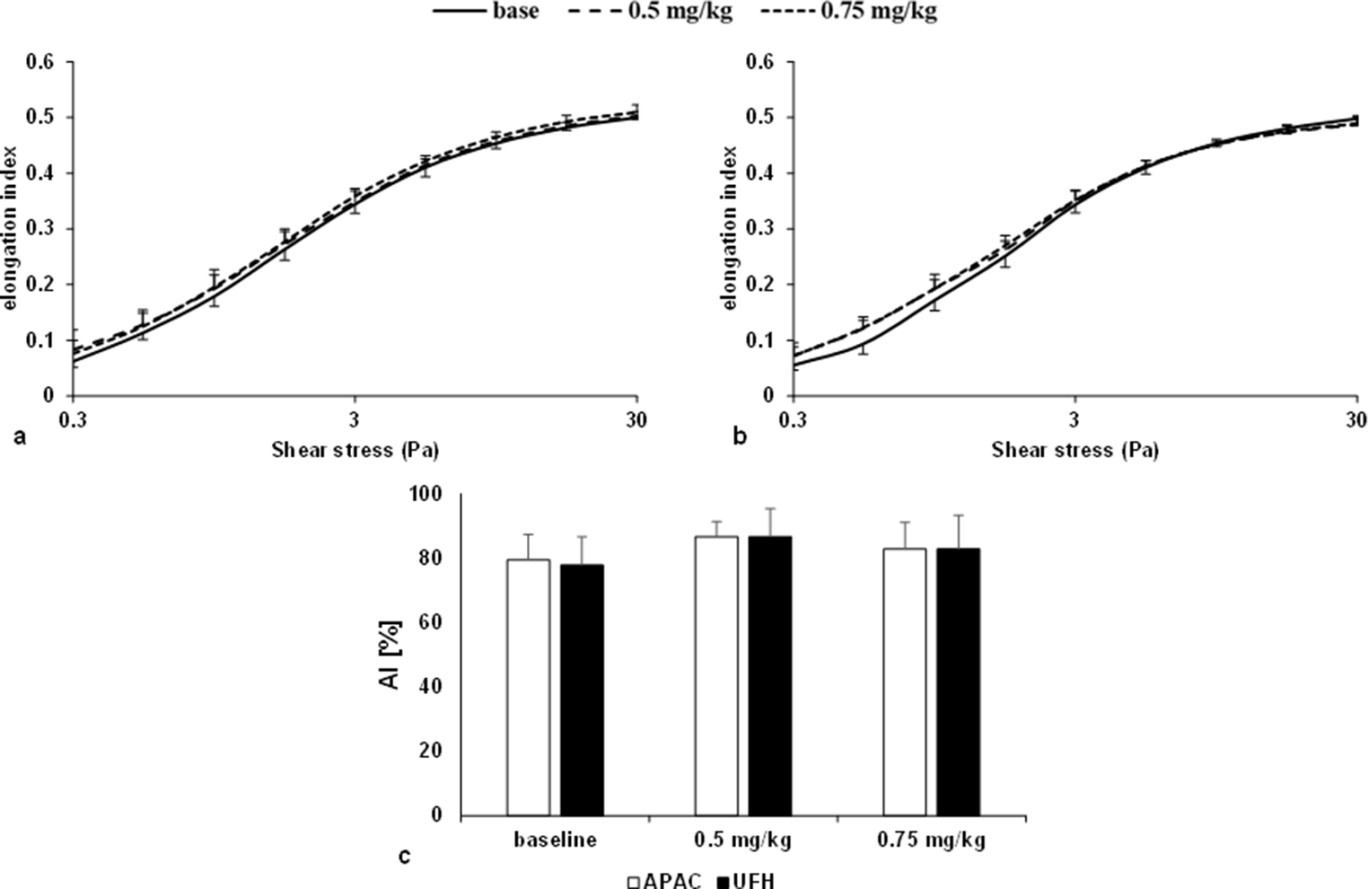
Elongation index in the function of shear stress (Pa) in UFH group (**a**) (*n* = 5) and APAC group (**b**) (*n* = 5) blood samples. Aggregation index (AI [%]) values measured after disaggregation (**c**)

Comparing the EI-SS curves, at low shear stress (< 3 Pa) both agents caused similar changes, with the values increasing. Conversely, at higher shear stress, a slight deterioration was observed in the APAC group, while an improvement was still observed in the UFH group. The red blood cell deformability representative curves of the UFH and APAC are shown in parts of *a* and *b* of Fig. 3. Bottom of Table 2 summarizes the data obtained by parameterization of the EI-SS curves.

Dosage of 0.75 mg/kg UFH caused a significant increase (*p* < 0.001 vs. baseline) in elongation index values measured at a shear stress of 3 Pa. The deterioration observed in the APAC group at the higher shear stress in the curves was also present the deterioration in the maximum elongation index (EImax) values. Both doses significantly decreased the EImax (0.5 mg/kg *p* = 0.024; 0.75 mg/kg *p* = 0.023 vs. baseline), indicating a deterioration in the RBC elongation at higher shear stress. The impairment caused by APAC at the high heparin 0.75 mg/kg was also significant compared to the same dose of UFH (approx. 135 IU/kg) (*p* = 0.009).

#### Red blood cell aggregation

A dose of 0.5 mg/kg of APAC and UFH slightly increased the rate of aggregation compared to baseline. This enhancement was not striking for the 0.75 mg/kg dose. As shown in part *c* of Fig. 3, similar changes were observed for both agents at the doses tested, which were not significant for either agent or dose.

## Discussion

The increased risk of bleeding complications associated with the combination of antithrombotic agents with different mechanisms of action, have raised interest in the development of cardiovascular therapies with dual antiplatelet and anticoagulant (APAC) activity. Using naturally produced heparin proteoglycans as a framework for biosynthetic alternatives, the protein conjugated UFH chains offers a promising route [14, 19, 21]. The micro-hemorheological properties of APAC did not differ from UFH, and these data do not refer to specific safety issues for this novel naturally occurring HEP-PG mimetic.

Our in vivo study showed that dual APAC in escalated bolus doses maintained the CBC relatively stable, but after protamine neutralization, some decrease of platelet count occurred. The dosing scheme in boluses 0.25 mg/kg to 0.5 mg/kg (approximately 45–90 IU/kg of heparin) was clinically relevant but the highest dose of 0.75 mg/kg exceeds the maximal dose allowance. We have unpublished data on the administration of APAC in healthy volunteers, which do not impact the CBC values (data in file).

Both APAC and UFH enhanced RBC aggregation. At a low shear stress, an improvement in red blood cell deformability was observed with both treatments, but at higher shear stress, red blood cell deformability deteriorated, which may have contributed to the increased RBC aggregation. Compared to heparin, APAC caused similar but minor microrheological changes that were not physiologically significant in vivo. Although some hematological variables were altered, they retained within biologically normal laboratory range for the species [30]. Heparin is widely used in the clinics for the treatment of thromboembolism and as an anticoagulant, for example during the extracorporeal circulation. Initial hyper-aggregation of RBC is observed in majority of patients with cardiovascular disease. The treatment with heparin, i.e., UFH or APAC in these patients could potentially affect blood rheology [7].

Previous studies have shown that APAC was reno-protective in acute ischemic kidney injury, in contrast to UFH [21]. We also have similar preliminary experimental data in myocardial infarction (submitted) and stroke [31]. APAC as a heparin proteoglycan mimetic inhibits coagulation initiated by the intrinsic coagulation pathway and thrombin, yet it differs from conventional heparins in inhibiting platelet aggregation and procoagulant activity [21]. Collagen-induced platelet aggregation is unaffected or even enhanced by UFH, whereas APAC attenuates platelet aggregation and deposition on collagen surfaces in a dose-dependent manner [13, 14, 21]. Platelet-induced thrombosis is prevented by APAC under high shear rates, whereas in the presence of UFH, these vessels are occluded. In a collagen-coated AV shunt model [14, 21], local administration of APAC reduced both platelet and fibrin deposition. PET scans have demonstrated that APAC targets and binds with a longer retention time to the injury site compared to UFH [19, 32].

Patients with effective acetylsalicylic acid (ASA) inhibition have been reported to have lower plasma fibrinogen level and red blood cell aggregation values compared to the ineffective acetylsalicylic acid medication (ASA resistance) [33]. The fractal dimension of erythrocytes decreases with increasing aspirin concentration, so the rate of aggregation decreases [34]. At the same time, increasing aspirin concentration and shear force increase the deformation index of red blood cells, i.e., their elongation capacity improves [34]. ASA together with APAC enhances the platelet aggregation inhibition potential, albeit APAC is not directly impacting the thromboxane pathway [19].

In our study, we found that RBC deformability improved with low shear stress, but the erythrocyte elongation index moderately decreased under higher shear stress. Still, increasing the dose of UFH or APAC did not affect this effect.

Heparin-induced red blood cell aggregation remains a complex and somewhat enigmatic phenomenon within the realm of medical science, posing challenges in terms of its therapeutic implications. While heparin, a commonly used anticoagulant, is generally considered beneficial for its antithrombotic properties, its apparent role in stimulating RBC aggregation raises intriguing questions and potential concerns. RBC aggregation is influenced by many factors such as hematocrit, free radicals, and fibrinogen levels [35–39]. The hemoconcentration measured with hematocrit here in pigs was small, although mathematically significant, not biologically so. Previous studies have found that a significant increase in RBC aggregation occurs at higher hematocrit levels, which also shows interspecific differences [35].

One notable indicator of this phenomenon is the elevation in the erythrocyte sedimentation rate (ESR), a parameter frequently used to gauge inflammation. The increase in ESR and low shear blood viscosity are often associated with increased RBC aggregation, and this observation is especially important for patients receiving heparin therapy [7]. Underlying health conditions that already influence RBC aggregation, i.e. sickle cell anemia or diabetes, could potentially have effects from heparin-induced RBC aggregation [40].

Moreover, the promotion of RBC aggregation by heparin can have broader implications in the realm of microcirculation. The microcirculatory system, consisting of tiny blood vessels and capillaries, plays a crucial role in tissue oxygenation and nutrient delivery. However, the alterations were numerically significant, but its biological effects are not clear. It is still not known where the ‘point’ is where micro-rheological changes turn to microcirculatory deterioration.

Heparin’s impact on blood viscosity and erythrocyte sedimentation rate has been the subject of extensive research, consistently revealing its influence on the aggregation RBCs across various concentrations [7]. The data consistently indicate a noteworthy trend of increasing blood viscosity and ESR levels, aligning with a concomitant rise in RBC aggregation. This phenomenon, while intriguing, raises concerns regarding the potential adverse effects of heparin on blood rheology.

An illustrative example of heparin’s influence is the observation that, in all examined donor blood samples, the average ESR surged by approximately 75% when the heparin concentration reached 100 U/ml [7]. Such significant changes in ESR are indicative of an alteration in RBC behavior, particularly their tendency to aggregate. These findings collectively point towards a negative effect of heparin on RBC aggregation, a factor of importance in understanding its clinical implications. The decrease of platelet count in the samples might be related to a ‘relative’ in vitro decrease, supposedly related to the role of heparin in altering the red blood cell surface properties [40].

Limitations of the study include the relatively low case number and the few selected dosages for comparison. The experiments were performed in juvenile pigs with healthy vasculature, and we plan to investigate changes in pigs with atherosclerosis in the future. The experiment was a short-term study with repeated bolus dosing, and we were targeted to detect acute alterations of hematologic and hemorheological variables.

## Conclusions

Our study is the first one to investigate the micro-rheological relation of the APAC administration. In the light of these data, it can be concluded that UFH and APAC, the dual antiplatelet and anticoagulant have similar micro-rheological properties. Despite the selective (collagen- and thrombin induced) antiplatelet effects, in addition to the traditional heparin-induced anticoagulant effects no signs of hemostatic impairment occurred, and the pharmacokinetic profile resembled earlier observations [26]. The use of a dual-acting formulation may be advantageous in patients who require concomitant antithrombotic agents with different mechanisms of action, to avoid the synergistic effects of the agents and increased risk of bleeding.
